# On the Advantages and Disadvantages of Inducing Premature Labour, with a View of Superseding Embryulcia, the Section of the Symphysis Pubis, and the Cesarean Operation

**Published:** 1801-01

**Authors:** James Barlow

**Affiliations:** Blackburn, Lancashire


### *To the Editors of the Medical and Phyſical Journal.*

GENTLEMEN,

SHOULD the annexed obſervations on a branch of Midwifery merit a place in the Medical and Phyſical Journal, they are at liberty for inſertion in your next Number. I am,

GENTLEMEN,

Your humble ſervant,

JAMES BARLOW.

*Blackburn, Lancaſhire,*
*Nov.* 20, 1800.

---

### *On the Advantages and Diſadvantages of inducing premature Labour, with a View of ſuperſeding Embryulcia, the Section of the Symphyſis Pubis, and the Ceſarean Operation.*

Nec Deus interſit niſi dignus vindice nodus. HORACE.

THE practice of exciting premature labour, in caſes where the pelvis is ſo much diſtorted as to render embryulcia neceſſary, has been recommended by various authors, though the exact limits preſcribed for its neceſſity have not hitherto been pointed out with that degree of preciſion which the importance of the operation demands; how far I have been able to illuſtrate the ſubject I cannot pretend to ſay: if, however, I am ſo fortunate as as to caſt a ſingle ray of light thereon, I ſhall not conſider my time miſpent.

To enter into a detail of all the various modes of delivery in caſes of deformity of the pelvis, would far exceed the limits preſcribed to the preſent undertaking; I will therefore only make a few animadverſions on the ſubject in queſtion, and wave entering into a laboured hiſtory of the operation, nor trouble my readers with aſcertaining the exact epoch which gave riſe thereto. The various gradations of deformity incident to the female pelvis, are frequently ſuch as involve the accoucheur in ſome degree of perplexity reſpecting the beſt mode of delivery to be adopted for the ſafety of both mother and child, and various are the reſources which obſtetric knowledge indicates for that purpoſe. The cruelty inflicted upon the fœtus by the uſe of the crochet, and the danger attendant upon the mother, are reaſons of ſufficient weight for the humane accoucheur to exerciſe his utmoſt ſkill in endeavouring to point out a more eligible expedient, in caſes of diſtorted pelvis, than that of the deſtruction of the fœtus; the deformity of the pelvis then, to a certain degree, is what has been conſidered by authors as an indication for bringing on premature labour, with a view of ſaving the life of the child, and leſſening the riſque of that of the mother.

On a ſuperficial view of the ſubject, it would appear to be pregnant with but few difficulties; but, on a more minute enquiry into the attendant circumſtances, we ſhall be compelled to acknowledge that many obſtacles preſent themſelves, ſome of which appear of conſiderable cogency; whether the removal of theſe lye within the ſphere of obſtetric art, or, otherwiſe, wait the interference of the legiſlature, time alone can only determine; it is nevertheleſs incumbent on every accoucheur (before he attempts the operation) to eſtabliſh in his own mind the morality of the practice, and exert his utmoſt ſkill, by every poſſible means, to extricate the environed fœtus from impending deſtruction.

This operation, however, can ſeldom be put in practice before it has been proved, by the event of a former labour, that the pelvis was ſo much diſtorted that the life of the child muſt have been inevitably deſtroyed before delivery could be accompliſhed; and as the human pelvis is liable by diſeaſe to become contracted into various ſhapes and degrees of diſtortion, from that of a well-formed pelvis to one ſo much contracted in its different apertures, that delivery per vias naturales becomes in ſome inſtances utterly impracticable. In theſe different ſhades of diſtortion, art has furniſhed us with means of accompliſhing delivery in various ways, according to the exigency gency of each individual deviation from the natural ftandard; but as authors, even in the prefent improved ftate of the art, differ in opinion on thefe important points of practice, it is to be hoped that every laudable endeavour to facilitate the difficulties attendant on parturition, will promote a free enquiry on the fubject, and ultimately have a tendency to the general good of fociety. To become acquainted with the fundamental principles of midwifery, and to afcertain the exact dimenfions of the pelvis, is a matter of the greateft moment to the accoucheur, before he determines on the operation; as want of this neceffary knowledge made by the touch, may involve the practitioner in irretrievable injury to the fœtus. The diftortions of the pelvis are generally induced by *rachitis* in the infantile ftate, or by *malacoftion* and *exoftofis* in the adult; it is thefe different caufes and degrees of diftortion which the accoucheur fhould keep in view when making the admeafurement per vaginam. When paffing the finger up the vagina, and any part of the os facrum, or lumbar vertebræ, be perceived to project into the cavity of the pelvis, or if the rami of the ifchia, or the fymphyfis of the os pubis, approach unufually near each other, we may conclude that fome degree of original mal-conformation exifts in the bones which compofe that aperture; yet it not unfrequently happens that the fuperior aperture is confiderably contracted, and the inferior cavity, or outlet, is even wider than natural: and to obtain a fufficient idea of the ftate of the apertures of the pelvis, in every direction, I have frequently found it neceffary to have recourfe to the paffing of the whole hand into the cavity of the pelvis; the three firft fingers of which are to be conducted to the brim, where the admeafurement may be generally afcertained to the fpace of a few lines, by placing them in different directions in the fuperior ftreight, and if, when kept clofe together, the fide of the fore finger touch the os pubis, and the third the projecting angle of the facrum, we may conclude that the fpace betwixt the two points, is manifeftly no more than two inches, an opening through which no living mature fœtus can poffibly be extracted alive; and when only two fingers can be placed in the fame manner, and betwixt the two angles above-mentioned, the accoucheur may reft fatisfied that, except a greater fpace can be obtained on either of the fides where the admeafurement is made, that no other mode of delivery, per vias naturales, can, with fafety to the mother, be adopted than either the crotchet or Cæfarean fection. This method of proceeding will generally furnifh the accoucheur with an accurate knowledge of the dimenfions of the pelvis, and direct him to the moft eligible refources in cafes of extreme deformity.

mity. In every inftance where I have had occafion to terminate the delivery with the crochet, it has been my conftant practice to afcertain the exact dimenfions in every poffible direction, that I might be armed with every requifite auxiliary in each fucceeding labour.

To thofe whofe opportunities of practice have not furnifhed them with a fufficient fhare of difcrimination by the touch per vaginam, to be enabled to afcertain to a tolerable degree of exactity the various fhades of diftortion which the female pelvis is liable to, I would recommend the ufe of the *calipers*, as an inftrument more eafily applied, and poffeffing fuperior advantages of accuracy over any other. See plate the 6th of Heath's Baudelocque; and for an account of their mode of application and utility, I will tranfcribe the 130th page from Vol. I. of that author.

"To determine how much the fuperior ftrait is defective in the aforefaid diameters, and meafure it by means of thefe compaffes, we take the thicknefs of the woman, from the middle of the mons veneris to the centre of the depreffion of the bafe of the facrum pofteriorly, by applying one of the points of the inftrument before, againft the fymphyfis of the pubis; and the other behind, a little under the fpine of the laft lumbar vertebra; and deduct three inches from that thicknefs in women that are thin, for the bafe of the facrum, and the anterior extremities of the offa pubis; the thicknefs of thefe latter being at moft but fix lines, and that of the bafe of the facrum two inches and a half; and fo conftantly fo, that I have not found a difference of a line in about five and thirty pelves, diftorted and contracted in all manner of ways, and in all poffible degrees.

"This fubtraction of three inches from the external thicknefs of the pelvis, in the faid directions, is alfo fufficient when the luftinefs is moderate; and we may add one or two lines more, when it is exceffive, becaufe the fat which forms the mons veneris, eafily fhrinks under the lenticular extremity of the leg of the compaffes. The refult of this procedure is fo exact, that the pelvis meafured with the common compaffes after opening the body, was not above a line over or under my eftimation in any one of my experiments. A greater precifion if we could obtain it, would be ufelefs, fince the choice of the moft proper methods for terminating the delivery in a given cafe, cannot depend on a line more or lefs in the diameter of the pelvis. According to thefe data, the knowledge of this diameter is eafily obtained. It is four inches when the external thicknefs of the pelvis meafures feven; it is but three when the latter only meafures fix; and but two when it does not exceed five, &c. I fuppofe the woman to be thin, as moft of thofe are who have been rickety."

In confidering the mechanical defcent of the fœtal cranium through the pelvis, it will appear manifeftly neceffary that the dimenfions of the one fhould bear fome mathematical proportion to the other; to obviate certain difficulties, Nature has wifely ordained a peculiarity of ftructure to the fœtal cranium, the mechanifm of which is fo formed, that the head of the fœtus is lefs compact than that of the adult, and the bones more loofe and numerous, confequently are better adapted for paffing through the pelvis, than if they had been firmly joined together.

The growth of the fœtus alfo varies very much during the different ftages of geftation; the embryo increafes more rapidly in fize during the firft weeks after conception, (than after it takes on the fœtal ftate, till the end of nine months,) though this increafe has various ftages of irregularity, till the full period of utero geftation. The walls of the pelvis are fo firmly connected together, and the change which the fœtal head is conftantly undergoing as geftation advances, will ever remain an obftacle to this fpecies of delivery, owing to the uncertain magnitude and degree of refiftance oppofed to the pelvis during its paffage through that tube. Without multiplying the various pofitions which the fœtal head affumes during the approach of labour, it may be neceffary to ftate, that the general and moft natural prefentation, is the vertex with the ears, nearly in a diagonal direction betwixt the pubis and facrum; the caufe of this part of the head defcending firft into the pelvis, may be owing in fome meafure to the foramen magnum being fituated nearer the occiput than the face, confequently is more mechanically inclined to become firft pufhed into the fuperior aperture of the pelvis; fometimes, however, the anterior fontenelle may at the commencement of labour be perceived to prefent in the axis of the fuperior ftrait, but this fmall deviation is generally rectified by the repeated action of the uterus, which forces the vertex into the axis of the pelvis, whilft the chin is bent down upon the breaft, and the face turned into the hollow of the facrum. If this procefs was not to take place, and the fontenelle was forced out of its proper direction, the labour would prove tedious, and probably might fometimes terminate in a face prefentation. The forceps and lever are inftruments well calculated for extracting the fœtus within certain bounds of difficulty, and claim a preference over every other invention, when ufed with a view of preferving the lives of both mother and fœtus; and when the fmall diameter of the fuperior ftrait of the pelvis offers a fpace

ſpace for the entrance of the head of the fœtus, equal to three inches in diameter, we may not altogether reject every hope of extracting a living mature fœtus through an aperture of the above ſpecified dimenſions, with either of theſe inſtruments; or if the child's head is ſmall, and not too firmly oſſified to elongate, and allow the bones to overlap by preſſure, and the forcible action of the uterus, we may ſometimes under theſe circumſtances meet with a favourable termination, when the diameter of the pelvis is rather under three inches. On the contrary, when the mother's pelvis is aſcertained to meaſure no more from pubis to ſacrum, or in any other of the apertures, than two inches, or two inches and a half, I am perſuaded that no mature fœtus can poſſibly be brought through a ſpace of theſe dimenſions, without deſtruction to the child; it is then in this intermediate degree of diſtortion, betwixt the poſſibility of delivery, (without injury to the mother or fœtus,) with the forceps or lever, and that ſhade of deformity which requires the application of the crotchet, where premature delivery ſeems moſt likely to become admiſſible. A queſtion then will naturally preſent itſelf to every accoucheur who deliberates on the ſubject, namely, to what degree of compreſſibility is the mature fœtal cranium capable of undergoing, without deſtruction to the child during its paſſage through the pelvis, and what analogy does it bear in computation with one of ſeven or eight months? The ſolidity of the fœtal head, and the re-action of the bones of the pelvis upon that body, are ſo variable in different children, that no exact criterion can be formed before birth; however, I will venture to riſk an opinion, that the diameter of the fœtal head, under certain ſituations, will bear a reduction in ſize by preſſure, from a quarter to half an inch, without producing much injury to the fœtus; but when the diameter of the head becomes much reduced beyond this, by either the forceps or lever, their application becomes dangerous to the child, and of courſe inadmiſſible on that ſcore. I conſider the fœtus a mere paſſive body during its paſſage through the pelvis; nevertheleſs, I am well aware that examples are not wanting, where the fœtal head has undergone a much greater reduction by compreſſure without proving fatal; however, I would have it underſtood, that what I have advanced, is what more generally takes place, and the deviations from the above rule are only exceptions therefrom. Some difference may reſult from the reduction of the volume of the cranium of a fœtus of eight, and one of nine months, as it will be generally allowed, that the bones which compoſe the cranium of the latter, will not admit of the ſame degree of reduction when compared to the former, yet, perhaps, a leſs a lefs variation in the fize of the head will take place betwixt the two, during their paffage through the pelvis, than what at firft would appear probable, owing to the greater power of action of the uterus during labour upon the body of a fœtus of nine months, than upon an immature one of feven or eight; and fome difference of refult may alfo arife to the fœtus from the length of time which may elapfe during the paffage of the head through the aperture of the pelvis.

The next queftion which occurs is, in what degree of diftortion of the pelvis it becomes neceffary to have recourfe to this operation? This point will not be eafily afcertained, even if the accoucheur was allowed to make an examination per vaginam; and as this is a matter not altogether attainable during geftation, it becomes requifite to ftate the circumfcribed dimenfions of the pelvis, wherein this operation is more likely to meet with fuccefs: I prefume then that a pelvis, the fmall diameter of which meafures from pubis to facrum about two inches or two inches and a half, appears to favour the fuccefs of this operation more than any other dimenfions; for, on the one hand, it is fufficiently evident that a mature fœtus cannot be born alive, when the dimenfions are under two inches and a half; and on the other, when the fhort diameter does not exceed two inches, the crotchet becomes neceffary; and fhould the fuperior aperture meafure only one inch and a quarter in the wideft part of its fuperior conjugate diameter, the only refource for the fafety of the mother and fœtus is the Cæfarean operation.

If it is proved that this operation cannot be performed with any degree of certainty of fuccefs to the fœtus, except where there is a fpace to be gained in fome part of the fuperior aperture, from two inches to two inches and a half, then it will follow that this mode of delivery muft be very much limited, infomuch, that a confiderable degree of hazard will always attend it, owing to the want of accuracy in difcriminating betwixt the relative fize of the fœtal head and the dimenfions of the pelvis. Having now pointed out the diameter of half an inch, as the moft warrantable fpace alloted for the fuccefs of this fpecies of operation, when performed at any time from the latter end of the feventh to that of the eighth month of utero geftation, before or after which periods, I think, no one juftifiable who induces premature labour under thefe circumftances; nor can I altogether give credit to thofe accounts, from whatever fource they may have been gleaned, where it is afferted that premature delivery has been fuccefsfully adopted when performed early in the feventh month of pregnancy, and where where the diameter of the pelvis meaſured not more than two inches in the wideſt part.

When this operation is had recourſe to, and the dimenſions of the pelvis are ſuch as promiſe ſucceſs, we ought to defer the attempt as near to that period fixed by Nature for the full evolution of the fœtus as circumſtances will admit, that thereby the child may acquire every poſſible advantage to enſure a healthy ſtate of exiſtence after birth. The period of eight months, or thereabouts, is the moſt advantageous time for the performance of this operation, when we conſider the precarious ſtate of a fœtus at a much earlier period; for I will venture to predict, that not one in twenty can ſurvive the birth ſo completely as to be reared, where labour is excited earlier than the ſeventh month of geſtation: no doubt, a few exceptions may be oppoſed to this general concluſion; but if we conſider the puny ſtate of theſe ſhort-lived individuals, and the miſery which their premature birth ſubjects them to, we ſhall have no cauſe to envy their ſituation; and few would prefer ſuch an ephemeral ſtate to a mere nonentity.

It is to be wiſhed, that authors who have had recourſe to this operation, had been more explicit in their accounts reſpecting the exact period of geſtation, and the premature fate of children, and how long they ſurvived that event; for it is not a ſufficient incitement to the operation, merely to bring a being into exiſtence which muſt inevitably periſh ſoon after birth. And this immature mode of delivery would conſequently be a means of ſubjecting the mother oftener to a ſtate of geſtation than if ſhe was allowed to complete the period of nine months. To obtain premature delivery, the membranes which envelope the fœtus are generally artificially ruptured prior to that event; and the time which elapſes before the uterus reſumes its expulſive efforts, are very variable, inſomuch that in ſome caſes even ſeveral days have paſſed before that organ has completed its evolution. When the waters are thus evacuated, and the fœtus left in cloſe contact with that viſcus during its repeated contractions, I am diſpoſed to conſider the life of the child, during its exit through the pelvis, involved in ſome danger, and that proportionable to the degree of diſtortion, and date of geſtation. It is not ſufficient that premature labour ſhould be indiſcriminately produced at a given period of geſtation, merely with a view of ſuperceding embryulcia, it is of more importance to look forward to the preſervation of the child; for every attempt made as early as the ſixth month of geſtation, either with the intent of avoiding embryulcia, or preſerving the life of the fœtus, will anſwer no other purpoſe than jugulare mortuos; for, as I have before obſerved, and what I wiſh further to to inculcate is, that every delivery excited by art, or otherwife cafually taking place at an earlier period of pregnancy than the feventh month, will generally prove deftructive to the fœtus, either during the time of delivery, or very foon after that event.

The fufficiency of augmentation of fpace gained by the fection of the fymphyfis pubis in extreme deformity of the pelvis, and the manifeft danger attending that operation, are circumftances fufficiently warrantable to preclude its ufe as a fubftitute for any of the above-mentioned modes of delivery. The Sigultian operation frequently involves the fœtus in imminent danger when the diftortion is confiderable; and in fome inftances where the fection has been made, the confolidation of the facrum and ilia have been fo firmly united, that no adequate fpace could be gained by a divifion of the fymphyfis pubis for the extraction of the child, and delivery has afterwards been obliged to be terminated by the crotchet or Cæfarean operation; and it has been doubted, and with fufficient reafon, whether the Sigaultian operation has ever been fuccefsful to either mother or child, in cafes where the fuperior aperture, from pubis to facrum, meafured lefs than two inches and a half; hence will appear the infufficiency of this operation, and the neceffity of its being banifhed in future from the practice of midwifery.*

It is a matter of fome confequence for the accoucheur to difcriminate, in cafes of diftortion of the pelvis, between exoftofis and malacoftion, as in the latter the bones will fometimes give way confiderably, either by the introduction of the hand, or the impulfe of extracting the child.

Two cafes of this fpecies of deformity have fallen under my care; in one of which, the projection of the lumbar vertebra and the connecting angle of the facrum were fo much bent into the cavity of the pelvis, that on the introduction of the finger up the vagina, a protuberance prefented to the touch, very much refembling the head of the fœtus, pretty far advanced into the pelvis; on carrying the finger a little higher up, paft the projection, I could afcertain the head of the child; but on moving the finger round the projecting part, the

---

* Amongft the numerous evils attending the Sigaultian operation, the laceration or feparation of the facro iliac fymphyfis is one of the moft dangerous, and to obviate which, and at the fame time allow a fufficient feparation of the fymphyfis pubis, appears no eafy matter; yet, to accomplifh which, I have however thought, that if a vice could be fixed externally upon the hips of the woman, with a fcrew fo managed, that during the operation and fubfequent treatment, no more fpace fhould be allowed to take place than what was fufficient for the extraction of the fœtus, by which means the injury in queftion would be in fome meafure obviated.

the diftortion was fo great, that the whole circumference, in any direction, did not exceed that of half-a-crown. This was on the 29th of April, 1792, at which time I delivered her with the crotchet, and the bones of the pelvis yielded confiderably to the impulfe of the head of the fœtus, which acted like a wedge during the efforts of extraction; yet, notwithftanding the pliability of the bones of the pelvis, and the debilitated ftate of her conftitution, fhe recovered fpeedily, and without interruption. On the 22d of February, 1794, I was in the neighbourhood where this poor woman refided, and hearing of her being ftill alive, I was led by curiofity to pay her a vifit, and if poffible, to obtain permiffion to examine her per vaginam, which was readily granted. I found the rami of the offa ifchia at their junction with the rami of the offa pubis overlapped each other, leaving a fmall opening under the fymphyfis pubis, fufficient to admit the finger to pafs into the vagina by that paffage, and another aperture below, but rather larger, and parallel with the tuberofities of the offa ifchia. From what I have been fince able to learn, fhe furvived this period near two years, at which time fhe was become fo crooked, that her breaft and knees were almoft in contact with each other, and after her death it was with difficulty fhe could be put into the coffin. This woman bore nine children, and died in the 39th year of her age: I attended her in the preceding labour to that abovementioned, which cafe was lingering and tedious; however, I fucceeded by terminating the delivery with the lever, and preferved the life of the child. The other cafe of malacofteon occurred to me in this town, about three years ago; the woman was in a dying ftate before I was confulted. On examination I found the pelvis very much diftorted, and fufpecting the deformity to arife from a ftate of malacofteon, I was induced, from the circumftances of the preceding cafe, to attempt delivery; and this more particularly, as the poor woman expreffed an earneft defire to be releafed from her prefent burthen, rather than die undelivered. On enquiry, I learned that fhe had only juft completed the feventh month of geftation; this ftage of prematurity induced me to attempt to turn the fœtus, and deliver by the feet, by which means I might give the child a greater chance of life; but with refpect to the mother, there was not the leaft profpect of her furviving delivery long. The os uteri being dilated, I accordingly introduced my hand, but the apertures of the pelvis were fo much diminifhed in their dimenfions, that it was with confiderable difficulty; however, I fucceeded, and delivered the woman of living twins; fhe died in about twelve hours after, and the children did not furvive their births many days. Having obtained leave to inſpect the body afterwards, I found the dimenſions and ſtructure of the pelvis as follow: The conjugate, or antero-poſterior diameter, from the ſymphyſis pubis to the projecting angle of the ſacrum, meaſured two inches and a half; and the antero-poſterior dimenſions on each ſide of the ſacro illiac ſymphyſis, between theſe two points, were, in ſome places, a few lines more than two inches and a half; from which it is obvious, that the form of the ſuperior aperture of this pelvis had a triangular appearance. The rami iſchii approached ſo near each other, that the ſpace left betwixt them would ſcarcely admit one finger; and on the introduction of the hand, when proceeding to turn the fœtuſes, theſe bones receded from each other very conſiderably; and the ſame effects were obſervable in the ſuperior ſtrait. The ſpongy nature of the bones of this pelvis were alſo manifeſted, by having very little cretaceous matter in their ſubſtance, and their texture was ſo ſoft, that they were eaſily cut with a knife. Many advantages, I am confident, may reſult from an accurate knowledge of the diſeaſes and ſtructure of the bones of the pelvis prior to delivery; it is, I am perſuaded, from a want of this information, that the Cæſarean operation has ſo often proved fatal in this country, for there is reaſon to ſuppoſe, that moſt of the women on whom that operation has been performed in this kingdom,* have been afflicted with malacoſteon, and conſequently in an irrecoverable ſtate, independent of any injury inflicted by the operation itſelf; and perhaps, if the nature of the diſeaſe had been more clearly underſtood, delivery per vias naturales might, in ſome inſtances, have been accompliſhed with ſafety to the child, without having recourſe to any other mode of delivery; and in theſe caſes, where malacoſteon has made ſuch dreadful ravages in the female conſtitution, beyond which the art of medicine has hitherto fallen ſhort of re-eſtabliſhing, it would be a nugatory practice, when attempted only with the view of preſerving the life of the mother, to have recourſe to either the Sigaultian operation or the Cæſarean ſection, as the woman would inevitably die, even if exempt from a ſtate of geſtation. If we advert to the numerous caſes of Cæſarean ſection performed on the continent, compared with thoſe of this country, it will be obvious, that the comparative fatality has been owing to this diſeaſe pre-exiſting to the operation; for, in no inſtance where the Cæſarean operation has been performed in this country, has it proved ſucceſsful to the woman, except the caſe of Jane Foſter, on whom† I performed the Cæſarean operation

* England, Ireland, and Scotland.

† Vide Medical Records and Reſearches, p. 154.

operation with fuccefs, at Blackrod, in this county, a few years fince; in which cafe the operation became neceffary, on account of a deformity of the pelvis, incurred by an accident prior to a ftate of geftation, and not from any conftitutional difeafe. The failure of fuccefs attendant on the Cæfarean operation betwixt this and a neighbouring nation, is more afcribable to conftitutional difeafe, which thofe women had laboured under in this country, who have undergone the operation, than to any difference of climate or mode of operating.

When premature delivery is attempted, with a view of fuperfeding the Cæfarean fection, it can only become neceffary when the dimenfions of the pelvis are fuch as preclude every other mode of delivery per vias naturales; upon this ground the practice will be confined to the embryon ftate, or at at leaft, foon after it has acquired to that of the fœtal period. The folicitude of the accouchenr in this extreme degree of diftortion of the aperture of the pelvis, is wholly directed to the prefervation of the life of the mother, as every endeavour within the limits of our art, when tending to preferve the fœtus in thefe fituations, will be abortive; and how far the morality of the practice may be juftifiable, when performed with the view of preferving the life of the mother, and facrificing that of the fœtus, is not a matter eafily determined; the well-known aptitude inherent in a woman to conceive under thefe circumftances, and the number of fœtufes eventually deftroyed with the intent of preferving the life of the parent, are incidents of the utmoft importance to the community, and claim a proportionate fhare of confideration from every accoucheur concerned on thefe unfortunate occafions. In a fituation fo depending, is the accoucheur excufable who tacitly complies with the requefts of the mother, and voluntarily facrifices a number of immature fœtufes with a view to her own prefervation? As the Cæfarean operation in this fituation may be made a matter of choice, may it not be allowable for the mother to exercife her judgment on the occafion? In certain cafes, where the diftortion of the pelvis is not very confiderable, it is a practice adopted by fome practitioners, as foon as the hand is admiffible into the uterus, to return the prefenting head of the fœtus, and deliver by the feet; how far this mode of practice may be juftifiable, I cannot pretend to fay, as I am at a lofs to conceive how any material advantage can be obtained by reverfing the head, except it be where the uterine efforts are too feeble to accomplifh the exit of the fœtus in a natural prefentation, and where the advantage gained arifes from the fuperior degree of power excited by the accoucheur over that of the uterus. In cafes of this nature, and where the diameter of the fuperior aperture meafures no more than three inches from pubis to facrum, the head of the fœtus will generally, by the repeated action of the uterus upon its body, be forced a certain diftance into the brim of the pelvis; when this is accomplifhed, and the head is afcertained to have advanced one-third within the fuperior aperture, it will be advifable to attempt delivery with the lever; for I have, in a few inftances, fucceeded with perfect fafety to both mother and child with this inftrument, even in cafes where the crotchet has been ufed in the preceding births; hence will appear the advantage of waiting till the head of the fœtus is become fo far advanced in the pelvis, that the lever is admiffible, rather than involving the life of the child in unneceffary danger, by the implicit act of turning and delivering by the feet; a practice, even in the moft promifing fituations, always attended with danger to the fœtus, and which has not hitherto been explicitly made clear by authors.

Another fituation wherein premature delivery has been adopted, is in cafes of habitual mifcarriages at a certain period of geftation, beyond which the fœtus is fuppofed to die. The practice of inducing premature labour while there remains a profpect of the fœtus being alive, will involve the accoucheur in much ambiguity, particularly as the exact date of impregnation cannot clearly be afcertained, nor can we altogether obtain a certitude of the life of the fœtus in utero, or whether the woman might not go to her full reckoning, till the art of midwifery has arrived at that acme of perfection, competent for the acquiring of the knowledge of thefe data. I think no one excufable who attempts this fpecies of delivery, as inftances are not wanting, where, after a number of periodical immature births, the woman has, at length, gone to the full period of nine months, and become mater familias.

Authors appear divided in opinion refpecting the danger incurred upon the mother, by exciting premature labour; the parity of hazard which refults from this fpecies of birth, on the fcore of the woman, at the period of feven or eight months, will vary according to circumftances, when contrafted with the event which will follow the termination of a labour at full time, and where the pelvis is diftorted to the degree which I have fixed upon as indicable for this operation.

Were we to form an opinion on the event of a mifcarriage at the abovementioned period, we might be led to conclude, that little danger to the mother would follow this mode of delivery; but as the uterus is an organ in no refpect governed by the will, and the efforts of that vifcus are moft regular when left uninterrupted, therefore no comparifon will hold good good betwixt a premature delivery and one artificially produced, nor betwixt one at the full period of pregnancy where the pelvis is well formed.

An objection of much weight againſt this operation will naturally impreſs the mind of every humane accoucheur, namely, the difficulty of ſecrecy by which premature labour is effected; for I am firmly perſuaded, if ever the method ſhould be divulged amongſt a certain claſs of individuals, it will ſoon become too generally known, and the abortive attempts will be innumerable; this will doubtleſs defeat the intention of the operation, and lead to a crime of a moſt cruel and inhuman nature.

